# Mechanistic Insights into Stereospecific Antifungal Activity of Chiral Fungicide Prothioconazole against *Fusarium oxysporum* F. sp. *cubense*

**DOI:** 10.3390/ijms23042352

**Published:** 2022-02-21

**Authors:** Xiaofang Yang, Ronggao Gong, Yuanqi Chu, Siwen Liu, Dandan Xiang, Chunyu Li

**Affiliations:** 1 Key Laboratory of Tropical and Subtropical Fruit Tree Research of Guangdong Province, Institution of Fruit Tree Research, Guangdong Academy of Agricultural Sciences, Guangzhou 510640, China; yangxf5220@163.com (X.Y.); sicauchuyuanqi@163.com (Y.C.); liusiwen@gdaas.cn (S.L.); 2 College of Horticulture, Sichuan Agricultural University, Chengdu 611130, China; 12800@sicau.edu.cn

**Keywords:** prothioconazole, enantiomer, Fusarium wilt of banana, *Foc* TR4, stereoselective

## Abstract

As a typical triazole fungicide, prothioconazole (Pro) has been used extensively due to its broad spectrum and high efficiency. However, as a racemic mixture of two enantiomers (*R*-Pro and *S*-Pro), the enantiomer-specific outcomes on the bioactivity have not been fully elucidated. Here, we investigate how chirality affects the activity and mechanism of action of Pro enantiomers on *Fusarium oxysporum* f. sp. *cubense* tropical race 4 (*Foc* TR4), the notorious virulent strain causing Fusarium wilt of banana (FWB). The Pro enantiomers were evaluated in vivo and in vitro with the aid of three bioassay methods for their fungicidal activities against TR4 and the results suggested that the fungicidal activities of Pro enantiomers are stereoselective in a dose-dependent manner with *R*-Pro making a major contribution to the treatment outcomes. We found that *R*-Pro led to more severe morphological changes and impairment in membrane integrity than *S*-Pro. *R*-Pro also led to the increase of more MDA contents and the reduction of more SOD and CAT activities compared with the control and *S*-Pro groups. Furthermore, the expression of Cytochrome P450 14α-sterol demethylases (CYP51), the target for triazole fungicides, was significantly increased upon treatment with *R*-Pro rather than *S*-Pro, at both transcriptional and translational levels; so were the activities of the Cytochrome P450 enzymes. In addition, surface plasmon resonance (SPR) and molecular docking illuminated the stereoselective interactions between the Pro enantiomers and CYP51 of TR4 at the target site, and *R*-Pro showed a better binding affinity with CYP51 than *S*-Pro. These results suggested an enantioselective mechanism of Pro against TR4, which may rely on the enantioselective damages to the fungal cell membrane and the enantiospecific CYP51 binding affinity. Taken together, our study shed some light on the mechanisms underlying the differential activities of the Pro enantiomers against TR4 and demonstrated that Pro can be used as a potential candidate in the treatment of FWB.

## 1. Introduction

Fusarium wilt of banana (FWB), caused by soil-borne fungus *Fusarium oxysporum* f. sp. *cubense* (*Foc*) [1,2,3], led to the destruction of banana Gros Michel (*Musa*-AAA) and still poses as the greatest biotic challenge to the successor banana Cavendish [4]. Four physiological races of *Foc* have been identified thus far based on the different banana cultivars they can invade. Among them, *Foc* tropical race 4 (TR4) is the most recently evolved and the most virulent strain. TR4 can infect a wide range of globally grown Cavendish cultivars [5] and has been spreading inexorably throughout the world’s banana-growing regions in recent years [6]. Multiple approaches have been attempted, including breeding for resistant cultivars, to tackle this disease, but none of them were successful enough to be taken to the next step in the field for effective control. Managing FWB is challenging due to *Foc*’s ability to survive in the soils for decades as chlamydospores [7]. Planting of resistant varieties is considered the most plausible option to manage FWB; however, the application of this approach is limited by consumers’ preference and may also lead to the de-emphasis of other approaches such as chemical control for long-term disease management [8]. Even though there are very few examples of clear progress for chemical control in the past 50 years, it is still an essential component of integrated management practices and has been proved to be effective in preventing the spread of *Foc* [9,10]. Prochloraz and propiconazole were reported to inhibit the mycelial growth of *Foc* significantly [10] but have not been used in the field by foliar spray. To expand the chemical control approach, more fungicides need to be screened, systematically investigated and then employed for FWB management. Triazoles have been used considerably as clinical drugs and as agricultural fungicides [11]. Most of the triazole fungicides are chiral containing one or several chiral centers and are commercialized in racemic mixtures [12]. Enantiomers of chiral triazoles usually behave enantioselectively, even though they have identical physicochemical properties. Often, the active enantiomer possesses activities against the target organisms, but the less active or inactive ingredients may display side effects and impose risks to nontarget organisms [13]. As a typical representative of triazole fungicides, prothioconazole (Pro) is very effective against a variety of plant pathogens, such as *Fusarium*, *Pyricularia grisea*, *Sclerotinia sclerotiorum*, *P. striiformis* on corn, legume crops and other economic crops [14,15]. It functions by inhibiting the Cytochrome P450 sterol 14*α*-demethylase (CYP51) enzyme that is involved in the biosynthesis of sterols in filamentous fungi [16,17]. Like most of the triazole fungicides, Pro has an asymmetrically substituted carbon atom and thus consists of a pair of enantiomers with confirmed configurations as *R*-Pro and *S*-Pro [18]. Pro is widely marketed and applied as the racemate. Significant stereoselective differences between Pro enantiomers were found in their environmental behaviors and toxicological effects. *R*-Pro is preferentially degraded in soils, and *S*-Pro is preferentially accumulated in earthworm (*Eisenia foetida*) [19,20]. Remarkable stereoselective endocrine-disrupting effects of Pro were found with the *S*-Pro, showing more potency than its *R*-enantiomers [21]. However, relatively few cases considered the potential enantioselective fungicidal activities. There is speculation as to whether Pro could also cause enantioselective fungicidal activities in TR4. A comprehensive understanding of the role of enantioselectivity of Pro in plant pathogen is very imperative for the application and regulation of this chiral compound.

We designed three bioassay experiments in vivo and in vitro to test the hypothesis that the Pro enantiomers behave enantioselectively in their fungicidal effects against TR4. Accordingly, the underlying mechanism for the enantioselective effects were further investigated by evaluating the morphological changes and impairment in membrane integrity, measuring the oxidative stress responses and testing the interactions with CYP51 in our study. The results will not only provide the basis for more accurate assessment of Pro activity, but also provide valuable clues for the rational design of novel fungicides as a strategy of *Foc* control.

## 2. Results and Discussion

### 2.1. Pro Stereoselectively Inhibits TR4 Growth and Impairs Its Pathogenicity

**Figure 1:**
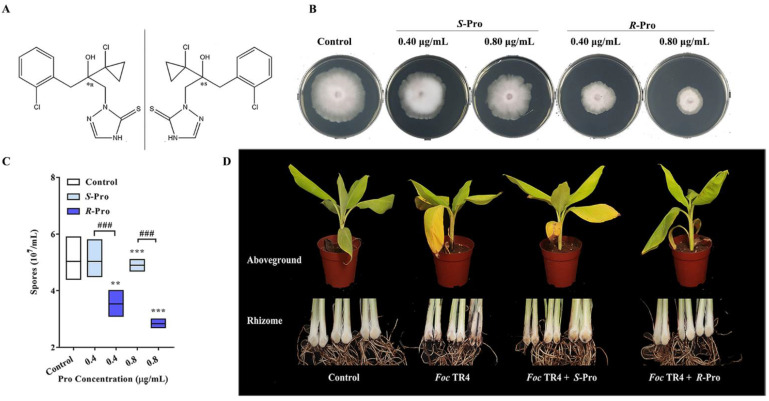
Pro enantiomers (**A**) inhibited the bioactivity of TR4 and impaired its pathogenicity stereoselectively. In *vitro* fungicidal activities of Pro enantiomers against TR4 in the mycelial growth (**B**) and spore germination assays (**C**), (**D**) Control efficacy of Pro enantiomers against TR4 on pot experiments. Data are shown as means ± SD (*n* = 3). ** *p* < 0.01 and *** *p* < 0.001 indicate significant differences between treatment groups and control group, ### *p* < 0.001 indicate significant differences between the *R*-Pro group and the *S*-Pro group.

To test the impacts of Pro enantiomers on the growth and the pathogenicity of TR4, both *S*-Pro and *R*-Pro were used (Figure 1A) to test their effects on the mycelia development (Figure 1B) and spore generation (Figure 1C) of TR4, and the symptoms induced by TR on banana seedlings (Figure 1D).

Pro enantiomers were shown here to inhibit mycelia formation of TR4 in a dose-dependent manner (Figure 1B). Under optimal conditions, *S*-Pro at 0.80 ug/mL slightly inhibited the mycelial growth of TR4 (Figure 1B), but *R*-Pro showed very significant inhibition of mycelia growth at both 0.40 ug/mL and 0.80 ug/mL (Figure 1B). The EC_50_ value of *R*-enantiomer against TR4 was 0.78 μg/mL (R^2^ = 0.9703), much higher than that of the *S*-enantiomer shown, with an EC_50_ value of 25.31 μg/mL (R^2^=0.9630). Therefore, the bioactivity of *R*-Pro was approximately 33 times higher than that of *S*-Pro.

Since spore germination is essential during the plant infection for fungal pathogens [22], the inhibition of spore germination was observed with the presence of Pro enantiomers. As shown in Figure 1C, the stereoselective inhibitions were also found among the Pro enantiomers. After 24 h incubation, the sporulation capacities of TR4 were significantly inhibited by *R*-Pro with both 0.4 ug/mL and 0.8 ug/mL concentrations, with a decrease of 36.90% (*p* < 0.001) and 49.40% (*p* < 0.001), respectively (Figure 1C). On the other hand, the sporulation capacity of TR4 was only slightly inhibited by *S*-Pro at 0.8 μg/mL with a decrease of 19.05% (*p* = 0.009) (Figure 1C).

Pot experiment was further conducted to find out whether Pro could endow the banana plants’ protective activities against TR4 stereoselectively. As shown in Figure 1D, normal symptoms of fusarium wilt, such as yellowing of the bottom leaves, upper leaf chlorosis and reddish-brown discoloration on the entire rhizome, were observed in plantlets inoculated with only TR4 or TR4 plus *S*-Pro. On the contrary, inoculation of TR4 together with *R*-Pro significantly delayed symptom development in the corms of banana plantlets. So, *R*-Pro showed a better protective effect than *S*-Pro on disease symptom development.

Taking together, these results indicated that there is enantioselective bioactivity between Pro enantiomers against TR4, and the antifungal activity of Pro may be attributed primarily to the *R*-enantiomer. Our results are in agreement with the fungicidal effects of other triazole fungicides. For instances, *R*-flutriafol and *R,S*-(+)-epoxiconazole were found to be the more effective stereoisomers with 1.49–6.23 and 1.30–7.25 times higher fungicidal activities than *S*-flutriafol and *S,R*-(-)-epoxiconazole, separately [23,24].

### 2.2. Pro Stereoselectively Leads to Morphological Changes and Impairment in Membrane Integrity in TR4

Since almost all the triazole fungicides are stated to act on the membranes of the fungal cells by altering their morphology or permeability [25], the changes of morphology and membrane integrity of TR4 in response to Pro enantiomers were evaluated.

**Figure 2:**
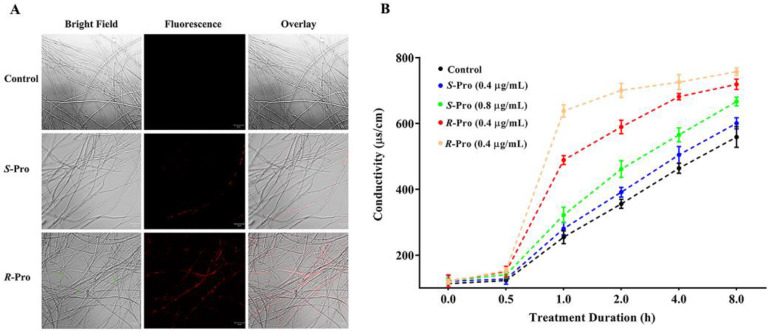
Pro stereoselectively led to morphological changes and impairment in membrane integrity in TR4. (**A**) Morphological changes and membrane integrity were evaluated using propidium iodide (PI) staining and visualized with a Zeiss LSM 710 scanning laser confocal microscope; green arrows indicate the morphological changes. (**B**) Time-course analysis of relative electric conductivity (REC) after treatment with Pro enantiomers for 0, 5, 10, 20, 40, 60, 80, 100, 120, 140, 160 and 180 min.

The mycelia were observed under a confocal laser scanning microscope after a 7 days incubation with DMSO (control) or Pro enantiomers. The control mycelia exhibited straight growth with smooth surface and clear septum (Figure 2A). *S*-Pro treatment resulted in slight twisting, while *R*-Pro caused significantly more granular aggregates and vague septum in the mycelia (Figure 2A). Hyphal septa are essential for the survival of fungi by sealing off damaged compartments from the viable mycelium, but cannot fully prevent the profound fungicidal activity, which takes effect in parallel in all hyphal compartments [26,27].

In this experiment, PI dye was fused to examine the membrane integrity of TR4, as PI is slightly larger than ATP (MW_PI_ = 668.41 g/mol; MW_ATP_ = 507.18 g/mol) and can only enter a cell when its membrane is damaged and open to at least 0.7 nm (https://bionumbers.hms.harvard.edu, accessed on 10 December 2021). As shown in Figure 2A, no detectable red-colored hyphae were observed in the control, confirming that cell membranes were intact. The red fluorescence, however, was observed in fungal cells upon the addition of *R*-Pro or *S*-Pro, indicating that Pro led to the disintegration of cell membranes. In addition, the effect was much more pronounced for *R*-Pro compared with the effect of *S*-Pro.

The membrane permeability for cations, considered as an important determinant of membrane integrity, stability and permeability [28], was further examined by the relative electric conductivity (REC). As shown in Figure 2B and Table 1, no significant change was found for all four Pro treatments at 0.5 h (*p* = 0.986, 0.369 and 0.117, 0.115 for 0.4, 0.8 μg/mL *S*-Pro and 0.4, 0.8 μg/mL *R*-Pro, respectively). However, when the treatment duration was extended beyond 0.5 h, the REC of tested TR4 increased with the Pro concentration and treatment time. *S*-Pro at 0.8 μg/mL showed a small but significant increase of the REC compared to the control from 1 h to 8 h treatment, whereas not at 0.4 ug/mL. On the other hand, *R*-Pro showed pronounced increases of REC with both 0.4 μg/mL and 0.8 μg/mL. The effect of Pro on the REC demonstrated an effective membrane disruption ability of Pro against TR4, which led to the leakage of intracellular electrolyte and increased conductivity [29]. It is worth noting that the speed of REC increase along with the treatment of R-Pro were more pronounced than *S*-Pro with the same concentrations.

### 2.3. Pro Stereoselectively Triggers Oxidative Stress Reactions in TR4

**Figure 3:**
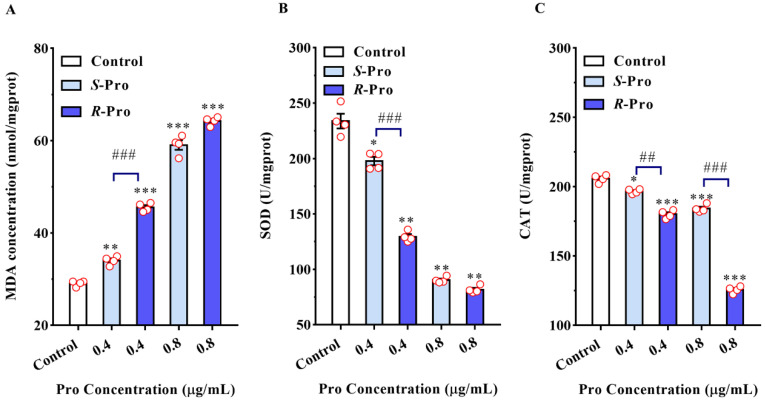
Pro stereoselectively triggered oxidative stress reactions in *Foc* TR4. Effects of Pro enantiomers treatment on Malondialdehyde (MDA) levels (**A**), Superoxide dismutase (SOD) (**B**) and Catalase (CAT) (**C**) activities. The results represent mean ± SD (*n* = 4). * *p* < 0.05, ** *p* < 0.01 and *** *p* < 0.001 indicate significant differences between treatment groups and control group; ## *p* < 0.01 and ### *p* < 0.001 indicate significant differences between *R*-Pro group and *S*-Pro group.

To further explore the cellular impact of Pro enantiomers on TR4, we measured the concentration of MDA, a common indicator of lipid peroxidation, oxidative stress, and subsequent cellular injury in cells and tissues [30,31]. The activities of two antioxidant enzymes, SOD and CAT, biomarkers of oxidative damage, were also measured. The MDA concentration was significantly elevated in both Pro enantiomers treatment groups compared to the control (*S-Pro*: *p* = 0.0019 for 0.4 μg/mL, *p* < 0.001 for 0.8 μg/mL; *R-Pro*: *p* < 0.001 for 0.4 μg/mL, *p* < 0.001 for μg/mL, respectively) (Figure 3A, Table 2). Moreover, *R*-Pro showed a noticeably higher increase than *S*-Pro did when both were used at 0.4 μg/mL (*p* < 0.001). By contrast, the activities of SOD and CAT were significantly decreased in both Pro enantiomer treatment groups relative to the control (*p* < 0.05). The SOD levels decreased by 44.67% (0.4 μg/mL) (*p* = 0.0011) and 65.06% (0.8 μg/mL) (*p* < 0.001) in *R*-Pro groups; and 15.44% (0.4 μg/mL) (*p* = 0.039) and 61.37% (0.8 μg/mL) (*p* < 0.001) in *S*-Pro groups. In addition, 0.4 and 0.8 μg/mL *R*-Pro resulted in significantly lower SOD activities compared to that in *S*-Pro groups by 34.56% (*p* < 0.001) and 9.55% (*p* < 0.001), respectively (Figure 3B, Table 2). The CAT levels decreased by 10.51% (0.4 μg/mL) (*p* < 0.001) and 38.95% (0.8 μg/mL) (*p* < 0.001) in *R*-Pro groups and 4.48% (0.4 μg/mL) (*p* = 0.018) and 12.53 (0.8 μg/mL) (*p* < 0.001) in *S*-Pro groups (Figure 3C, Table 2). In addition, 0.4 and 0.8 μg/mL *R*-Pro resulted in significantly lower CAT activities compared to that in *S*-Pro groups by 8.43% (*p* = 0.0016) and 31.78% (*p* < 0.001), respectively (Figure 3C, Table 2).

Our results indicated that Pro-induced fungicidal activity against TR4 was closely associated with elevated contents of MDA, and decreased activities of SOD and CAT. MDA has been frequently considered as an indicator of lipid peroxidation whose production can exacerbate membrane injury and cell senescence [32,33]. Our examination of MDA upon treatment with Pro enantiomers provided further evidence that cell damage and perturbation of the bilayer membrane structure led to decreased fluidity, increased permeability, and disturbance in phospholipids in TR4 cells, which was consistent with the damage of cell membrane observed by increased REC and PI influx [34]. Similar results had been obtained by Lee et al., who found that dicarboximide fungicides caused significant lipid peroxidation by increasing the MDA contents in a concentration-dependent manner in *Botrytis cinerea* [35]. SOD and CAT are enzymes constituting the primary defense system against reactive oxygen species by catalyzing the dismutation of O_2_^−^ to H_2_O_2_ and promoting the further conversion of H_2_O_2_ into oxygen and water in a coordinated manner [36]. In the present study, the activities of CAT and SOD enzymes were damaged upon the exposure of Pro, leading to the diminutions of the contents of CAT and SOD. High activities of these two enzymes are beneficial to alleviate oxidative stress during their storage. Therefore, the decreases of CAT and SOD demonstrated that the oxidative stress effect was induced by Pro enantiomers in TR4 that is often characteristic of fungal responses to stress [37]. Moreover, both the enantiomers can cause oxidative stress effects, and the oxidative injury caused by *R*-Pro was more noticeable. With these results, we can conclude that, in comparison with *S*-Pro, *R*-Pro inhibited the mycelial growth and destroyed the membrane integrity of TR4 more potently.

### 2.4. Pro Stereoselectively Upregulates the Expression of CYP51 in TR4

**Figure 4:**
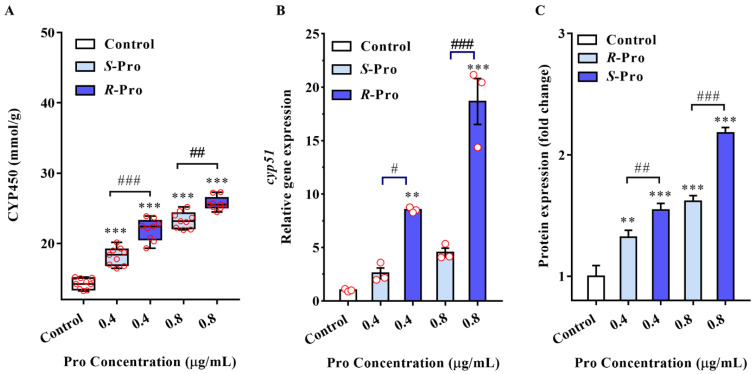
Pro stereoselectively induced the expression and quantitative of CYP51 in TR4. (**A**) Enzyme activity of CYP51; (**B**) Quantitative PCR relative expression levels of CYP51 mRNA transcripts, the *cyp51* transcript levels were in terms of 2^−ΔΔCt^ values and normalized to *g6dh* reference gene levels; (**C**) Protein expression for CYP51 by Western blot. The expression levels of *cyp51* gene and protein were detected with RT-PCR and Western blot and normalized with respect to *g6dh* and *β-actin*, respectively. The results represent mean ± SD (*n* > 3). ** *p* < 0.01 and *** *p* < 0.001 indicate significant differences between treatment groups and control group; # *p* <0.05, ## *p* < 0.01 and ### *p* < 0.001 indicate significant differences between *R*-Pro group and *S*-Pro group.

CYP51 performs imperative roles in the primary and secondary metabolic pathways, in the metabolism of numerous xenobiotics consisting of pesticides, and serves as the ubiquitous target of fungicides [38]. To explore the mode of actions of Pro enantiomers at the molecular level, we examined if the enantioselective effects observed in our study are related to the stereoselective interactions between the Pro enantiomers and CYP51. TR4 has three *cyp51* genes, FOIG_04112, FOIG_00890, and FOIG_10483, among which, FOIG_00890 shares 98.67% amino acid sequence identity with CYP51B from Fusarium musa [39]. Meanwhile, the CYP51B is expressed constitutively and considered to be primarily responsible for sterol 14 α-demethylation encoding in all sequenced filamentous fungi. Therefore, FOIG_00890 was chosen in our study to explore the interactions between the Pro enantiomers and CYP51 [40,41]. The effects of Pro enantiomers on Cytochrome P450 activities were analyzed first (Figure 4). We observed that Cytochrome P450 enzyme activities were strongly stimulated by both S-Pro and R-Pro at both 0.4 μg /mL and 0.8 μg/mL (*p* < 0.001) (Figure 4A). In addition, the stimulation effect of *R*-Pro was greater than that of *S*-Pro (*p* < 0.01 and *p* = 0.0022, respectively; Figure 4A). We then examined the transcript and protein levels of CYP51. The relative expression of *cyp51* gene was induced 8.52 (*p* = 0.0026) and 18.67 (*p* < 0.001) times by 0.4 and 0.8 μg/mL of *R*-Pro, respectively (Figure 4B). The mean relative expressions of *cyp51* induced by *R*-Pro were significantly higher compared to that by *S*-Pro (*p* = 0.013 and *p* < 0.001 for 0.4 and 0.8 μg/mL, respectively, Figure 4B). The expressions of CYP51 proteins upon the addition of Pro followed a trend comparable to the pattern of the *cyp51* gene expressions, increased progressively from the control to 0.4 ug/mL of *R*-Pro and *S*-Pro then to 0.8 ug/mL of *R*-Pro and *S*-Pro (Figure 4C). Being utilized as the main target of triazole fungicides, CYP51 is widely distributed in the fungal kingdom and inhibited by the triazole fungicides [16]. Alternatively, the increased expressions of CYP51 in our study might be a compensatory effect to furnish protection for TR4 from Pro by weakening the intrinsic activity which is identical to one of the mechanisms responsible for the resistance against triazoles [42]. Since CYP51 is an endogenous signaling molecule, an evaluated metabolism burden induced by Pro enantiomers may impose destructive perturbations in the normal functions of TR4. The inducible expression of CYP51 may not provide an explanation for the molecular mechanism of Pro uses for its control of TR4 directly; however, it demonstrated that Pro may set off the defense responses of this pathogen.

### 2.5. The Molecular Stereoselective Interactions between the Pro Enantiomers and CYP51 of TR4

It is known that Pro enantiomers interact directly with the host target CYP51 [43,44], but no corresponding structural data exist to date on this interaction yet [45]. In addition to the physiological and biochemical changes induced by Pro in TR4 cells, we are also interested in how Pro enantiomers may alter these responses of TR4 stereoselectively. To address this, the underlying binding potencies and interactions of Pro enantiomers with the CYP51 protein (N1RIS1_FUSC4) were further analyzed using the SPR biosensor and molecular modeling, respectively.

**Figure 5:**
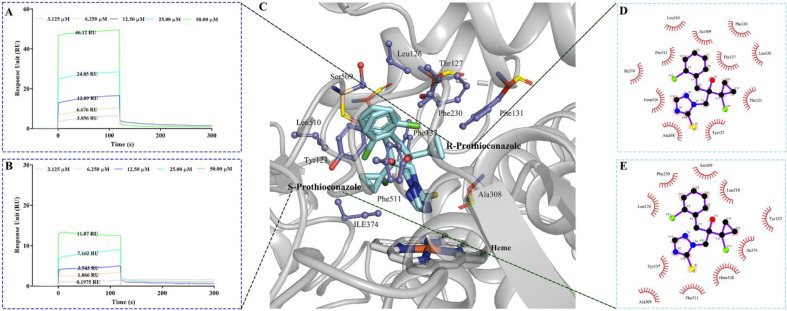
The molecular stereoselective interactions between the Pro enantiomers and CYP51 of TR4. (**A**,**B**) Binding of *R*-Pro (**A**) and *S*-Pro (**B**) to captured His-tagged N1RIS1_FUSC4 measured by surface plasmon resonance (SPR). Colored lines represent experimentally recorded values at different concentrations. Binding affinities were determined by kinetic analysis using one binding site model, used for fitting of SPR data. The sensorgrams shown are representative from three independent experiments; (**C**–**E**) Molecular docking analysis of Pro enantiomers. (**C**) Pose view of interactions of Pro enantiomers with CYP51, 2D diagrams of the interactions between the CYP51 and the two isomers: (**D**) *R*-Pro and (**E**) *S*-Pro.

First, recombinant N1RIS1_FUSC4 protein was purified from *E. coli* and confirmed by SDS/PAGE analysis. As predicted, the purified protein had a molecular weight of 54 kDa with a ≥90% purity (Figure S2). To test the binding affinities, N1RIS1_FUSC4 was immobilized on the CM5 sensor chip by the standard amine-coupling procedure with an immobilization level of approximately 8000 resonance units (RU) for the SPR study. Pro enantiomers were diluted into the running buffer at concentrations ranging from 3.125 to 50.00 μM (3.125, 6.250, 12.50, 25.00 and 50.00 μM) and injected, respectively, for detailed kinetic analysis. As shown in Figure 5A,B, the SPR experiment revealed quite efficient binding of Pro to N1RIS1_FUSC4. Interestingly, similar trends were observed for both the enantiomers that the response units (RU) of SPR detection increased in clear concentration-dependent manners. However, the SPR sensorgram indicated that the binding capacity of *R*-Pro to N1RIS1_FUSC4 was higher than the binding capacity of *R*-Pro to this protein at same concentration. Furthermore, the binding kinetic constants were fitted using reversible 1:1 bimolecular interaction model supplied in the Biacore software. The corresponding KD (equilibrium dissociation constant) values were found to be 3.42 × 10^−10^ M (Ka = 8.09 × 10^6^ M^−1^s^−1^, Kd = 2.76 × 10^−3^ s^−1^) and 4.82 × 10^−8^ M (Ka = 4.74 × 10^5^ M^−1^s^−1^, Kd = 2.28 × 10^−3^ s^−1^) for *R*-Pro and *S*-Pro to N1RIS1_FUSC4, respectively. The kinetic analyses revealed that *R*-Pro associated with CYP51 with faster on-rate, and dissociates with almost the same off-rate, than *S*-Pro. SPR analysis offered more novel insights into the binding properties (association and dissociation) between CYP51 and the two enantiomers.

Next, homology modeling and molecular docking procedures were conducted based on the SPR affinity measurements to further understand and characterize the different interactions between this pair of enantiomers with CYP51. A structural model of the CYP51 protein from TR4 was built by homology modeling using structures of 6CR2, 5V5Z, 5JLC and 5ESI as templates (Figure 5C and Figure S3). The subsequent docking studies with the CYP51 model revealed that both the enantiomers stretched into the same hydrophobic pocket formed by Tyr137, Phe131, Phe230 and Phe511 and were bound close to the heme group (R:2.99 Å and 71.07°; S:2.97 Å and 77.75°) in the active site of CYP51 by interacting with active site residues such as Leu126, Tyr137, Phe230, Ala308, Ile374, Ser509, Leu510 and Phe511 (Figure 5C) [41,46]. The benzene ring and triazole ring preferred the same orientations, while the cyclopropane and two chlorine atoms showed the opposite binding orientations for this pair of enantiomers (Figure 5C). In addition, *R*-Pro bound to residues Thr127 and Phe131 with a binding affinity of −8.07 kcal/mol, whereas *S*-Pro bound to residue Tyr123 with a binding affinity of −7.89 kcal/mol (Figure 5D,E). Thus, the SPR and docking assays seem to support our hypothesis that Pro has enantioselective binding interactions with its target CYP51 protein both in binding affinity and in silico modeling, and *R*-Pro possesses much greater effects on TR4 than *S*-Pro due to its stronger binding affinity to CYP51.

## 3. Materials and Method

### 3.1. Chemicals and Reagents

Pro ((R,S)-2-[(2-(1-chlorocyclopropyl)-3-(2-chlorophenyl)-2-hydroxypropyl]-1,2-dihydro-3H-1,2,4-triazole-3-thione), the enantiomers of pro (*R*- Pro and *S*-Pro) (≥98.0% purity), were prepared by Chiralway Biotech Co., Ltd. (Shanghai, China). The two enantiomers are mirror images of each other (Figure 1A). Stock solutions of these chemicals (1 mg/mL) were prepared in DMSO and stored at 4 °C.

### 3.2. Fungal Strains Culture Conditions and Experimental Design

The pathogenic TR4 strain II5 (VCG01213) used in this study was maintained in PDA (potato dextrose agar medium, Guangdong Huankai Microbial SCI. & Tech. Co. LTD., Guangzhou, China) with 15.0% glycerol at −80 °C in the Institute of Fruit Tree Research, Guangdong Academy of Agricultural Sciences. The fungal strain was routinely inoculated onto the center of petri plates (90 mm diameter) and incubated at 28 °C for 7 days and the fresh cultures were used to prepare inoculums for experiments. The bioactivity of *rac*-Pro against TR4 was determined with EC_50_ in our preliminary experiment and the corresponding EC_50_ (0.8 μg/mL) and 1/2 EC_50_ (0.4 μg/mL) (Text S1, Figure S1) concentrations were chosen as experimental concentrations in this work.

### 3.3. Stereoselective Fungicidal Activity of Pro Enantiomers against TR4

The stereoselective fungicidal activity of Pro enantiomers against TR4 were determined with three different methods. First, the mycelial growth inhibition method was used to assess the sensitivity of TR4 on fungicide-amended PDA as described in Text S1 and the EC_50_ was calculated using GraphPad Prism 7 (GraphPad Software Inc.; San Diego, CA, USA). Second, the conidial germination inhibition assay was conducted using the concavity slide method [47]. Briefly, fungal conidia were collected from PDA plates after 7 days of incubation at 25 °C in the dark, washed with 5 mL sterile water and filtered through by 4 layers of lens papers. Then, 50 μL spore suspensions (1.0 × 10^7^ conidia/mL) containing different concentrations of Pro enantiomers (0.4 and 0.8 μg/mL) were placed on slides and cultivated at 25 °C in the dark for 24 h. Suspensions with equal amount of DMSO served as controls. The total number of conidia in 3 randomly chosen areas was counted on each slide under a light microscope (Carl Zeiss, Jena, Germany) and the inhibition rates of conidia germination were calculated. All experiments were repeated twice with 3 replicates. For the third method, the in vivo stereoselective efficacy of Pro enantiomers against TR4 were tested using pot experiment with Baxi (AAA, susceptible) banana plantlets in the greenhouse. Briefly, the spore suspensions were prepared into 1 × 10^8^ conidia/mL containing 1 μg/mL Pro enantiomers or DMSO by sterile water with Triton 100 (0.1% *v/v*). Banana plantlets (*n* = 10) were surface-sterilized with 75% ethanol, rinsed with sterile distilled water for 3 times, root-immersed in spore suspensions for 24 h and then planted in a greenhouse. The plants were examined, and symptoms were recorded 21 days post inoculation.

### 3.4. Morphology and Propidium Iodide (PI) Influx of TR4

The effects of Pro enantiomers on the morphology and membrane integrity of TR4 were assayed with propidium iodide staining assay method. TR4 produced on PDA amended with Pro enantiomers at 0.4 and 0.8 μg/mL were harvested after incubating at 28 °C for 7 days, resuspended in phosphate-buffered saline (PBS) solution (pH = 7.4) and added with PI at the final concentration of 10 μg/mL. TR4 were centrifuged at 1500 rpm for 5 min after incubating with PI at 30 °C for 5 min in the dark and washed twice with PBS solution (pH = 7.4) to remove residual PI. The images were captured with a Zeiss LSM 710 scanning laser confocal microscope (Carl Zeiss, Jena, Germany).

### 3.5. Cell Membrane Permeability of TR4

Electrolyte leakages of TR4 induced by Pro enantiomers were measured according to a previously described method with minor modifications [48]. Fresh mycelia (0.5 g) were harvested from PDB after a 7-day incubation and washed twice with sterile deionized water and then suspended in 20 mL sterile deionized water containing different concentrations of Pro enantiomers (0, 0.4 and 0.8 μg/mL). The electrical conductivity of each suspension was measured with a conductivity tester (Sangon Biotech, Shanghai, China) at 0, 5, 10, 20, 40, 60, 80, 100, 120, 140, 160 and 180 min, respectively, and the suspensions were boiled for 5 min after the 180 min incubation to measure the final conductivity. The relative conductivity of mycelia was calculated as the following formula: relative electric conductivity (REC) (%) = conductivity/final conductivity × 100. There were three replicates for each treatment and the experiment was repeated independently twice.

### 3.6. Determination of Cellular Enzymatic Activity

TR4 were harvested (0.5 g) and homogenized in 5 mL PBS solution (pH = 7.4). The supernatants were collected and used for measurement after being centrifuged at 10,000× *g* for 10 min. The malondialdehyde (MDA) contents were determined with MDA assay kit (Jianglai Biotechnology Co., Ltd., Shanghai, China) according to the manufacturer’s instructions. The detections of SOD and CAT activities were measured using a Total Superoxide Dismutase (T-SOD) Assay Kit and a Catalase (CAT) Assay Kit (Grace Biotechnology Co., Ltd., Suzhou, China). One unit of SOD is defined as the amount of enzyme inhibiting the rate of nitrite ion generation by 50% as monitored at 550 nm. The CAT activity was measured by analyzing the rate at which it caused the decomposition of H_2_O_2_ at 405 nm and one unit of CAT activity was defined as the amount of enzyme required to consume 1 μmol H_2_O_2_ every minute.

### 3.7. Real-Time PCR

The expression levels of genes in TR4 under the stress of Pro enantiomers were analyzed. About 1 × 10^6^ conidia were used to inoculate 100 mL of liquid PDB (potato dextrose broth, Guangdong Huankai Microbial SCI. & Tech. Co. Ltd., Guangzhou, China) that was supplemented with DMSO as control or Pro enantiomers at 0.4 and 0.8 μg/mL (0.5% DMSO, *v/v*). The mycelium was harvested from each culture (4 per condition) after 3-day incubation in the dark (28 °C, 120 rpm) and quickly washed with sterile water before being flash-frozen in liquid nitrogen and ground with a mortar and pestle. RNA extraction was performed on a 100 mg of sample using the TRIzol Reagent (Takara, Dalian, China) according to the manufacturer’s instructions. Detailed procedures of RNA extraction, purification, and quantification are provided in SI (Text S2). The primer sequences of the selected genes were obtained by using the online Primer 3 program (http://frodo.wi.mit.edu/, accessed on 15 January 2022; Table S1), and *g6dh* was used as the endogenous reference gene to normalize the gene expression levels. The RT-PCR was carried out on an ABI 7300 device (PerkinElmer, Applied Biosystems, Foster City, CA, USA) and the different transcription levels were calculated using 2^−ΔΔCt^ method [49].

### 3.8. Western Blot Analysis

Fresh mycelia (200 mg) of each culture treated with DMSO or different concentrations of Pro enantiomers were harvested, finely ground and suspended in 1 mL of extraction buffer (50 mM Tris-HCl, 100 mM NaCl, 5 mM EDTA, 1% Triton X-100, 2 mM phenylmethylsulfonylfluoride (PMSF), pH 7.5) containing 10 μL of protease inhibitor cocktail (Sangon Co., Shanghai, China) and homogenized with a vortex shaker. Then the supernatants were collected after the lysate was centrifuged at 10,000× *g* for 20 min at 4 °C. The protein concentration was determined using the BCA protein assay kit (Keygen, Nanjing, China). Equal amounts of protein (20 μg) were separated by 10% sodium dodecyl sulfate-polyacrylamide gel electrophoresis (SDS-PAGE) and transferred to polyvinylidene difluoride filters (PVDF, BioRad, Hercules, CA, U.S.A). The CYP51 protein was detected using the CYP51A1 antibody (1:1000, Abcam, Cambridge, MA, USA) overnight at 4 °C after 1 h blocking with 5% non-fat milk in TBST and then with secondary antibody (1:5000) for 2 h at room temperature. Blots were developed with the ECL system (GE Healthcare). Equal loading of protein is normalized by β-actin as an internal standard. Band intensity was quantified by densitometry using Image J program (Image Processing and Analysis in Java; NIH, Bethesda, MD, USA).

### 3.9. Direct Binding Assays between CYP51 and Pro Enantiomers

The recombinant N1RIS1_FUSC4 was heterologously expressed in *Escherichia coli* (*E. coli*) and purified with Ni^+^ columns (HisGraviTrap™ HP column, GE Healthcare, Uppsala, Sweden) following the manufacturer’s instructions (Text S3). The recombinant CYP51 protein from TR4 (Eburicol 14-alpha-demethylase, N1RIS1_FUSC4, https://www.uniprot.org/uniprot/N1RIS1 accessed on 3 February 2022) was immobilized on a CM5 sensor chip (GE Healthcare surface) using the standard amine-coupling procedure, according to the manufacturer′s guidelines. The binding affinities and kinetics of Pro enantiomers with N1RIS1_FUSC4 were performed at 25 °C with a Biacore T200 instrument (GE Healthcare) according to a previous procedure (Text S4) [50].

### 3.10. Homology Modeling and Molecular Docking

The CYP51 structure of TR4 was modeled based on the crystal structures of 5V5Z [51], 5JLC [51] and 5ESI [52] through multi-template modeling using the Modeller 9.20 [53] and AutoDock Vina [29]. A total of 20 structures were constructed and the one with the least Discrete Optimized Protein Energy (DOPE) score was selected for the docking assays. The quality of selected 3D model was assessed using “Verify Protein (Profiles-3D)” protocol of Discovery studio (DS) and the evaluation and analyses of its structure and stereochemical were performed using ProSA-web Z-scores (https://prosa.services.came.sbg.ac.at/prosa.php, accessed on 15 January 2022). Energy minimization of the protein structure was performed by applying the “prepare protein” protocol of DS by adding missing atoms, inserting missing loops, assigning charges and fixing CHARMm forcefields. A grid box (15 × 15 × 15 Å) of CYP51 was predicted using the “Define and Edit Binding Site” protocol of DS. Possible binding modes between Pro enantiomers and CYP51 model were carried out using the DS Visualizer Pro and the Dock ligands (CDOCKER) protocol, respectively. Ten docked conformations for each enantiomer were scored according to a free energy cost function, and the first ranked conformation was selected for analysis.

### 3.11. Data Processing

SPSS 20.0 (V.26.0, SPSS Inc., Chicago, IL, USA) was used for statistical analysis. One-way analysis of variance (ANOVA) followed by Dunnett’s *t*-test was employed to analyze the differences between the control and experimental groups. Statistical significance was set at *p* < 0.05 and increasing levels of confidence are displayed as *p* < 0.05, *p* < 0.01 and *p* < 0.001. All results are shown as mean ± standard deviation (SD). Graphs presented were prepared via GraphPad Prism 7.

## 4. Conclusions

Our study shows that both Pro enantiomers could exhibit fungicidal activities against TR4 by modifying the mycelial morphology, damaging the integrity of cell membrane and inhibiting the activities of the major antioxidant enzymes. In addition, the bioactivity of Pro shows strong enantioselectivity, with *R*-Pro exerting more potent fungicidal activity than *S*-Pro. The enantioselective induction of CYP51 (enzyme activity, gene and protein expressions), to a certain extent, seems responsible for the cellular mechanisms of the enantioselective fungicidal activity. The affinity values and the structural information obtained from the SPR biosensor technique and molecular docking further elucidate the enantioselective interactions between Pro enantiomers and CYP51, which provides new insights into the molecular mechanism of the Pro enantiomers against TR4. These results will not only provide the basis for more accurate assessment of the application of Pro chemicals, but also provide valuable clues for the discovery and rational design of novel fungicides as a strategy of *Foc* control. Furthermore, as a large number of fungicides work similarly by targeting CYP51, the enantioselective fungicidal activity of Pro may be extended to other fungicides. Our results demonstrate the need to take the enantioselectivity into account for comprehensive risk assessments during fungal treatment for plant disease control.

## Figures and Tables

**Table 1:** Cell membrane permeability of TR4 in terms of relative electric conductivity (REC) after treatment with Pro enantiomers.

Time (h)	Control	Pro Enantiomers (μg/mL)			
		*S*-Pro		*R*-Pro	
		0.4	0.8	0.4	0.8
0	15.15 ± 1.42	16.04 ± 2.40	16.37 ± 1.17	16.16 ± 2.42	15.49 ± 0.94
0.5	16.35 ± 0.86	16.98 ± 2.08	18.82 ± 1.32	19.88 ± 2.19	19.90 ± 0.95
1.0	33.94 ± 2.59	37.34 ± 2.53	43.13 ± 3.16 **	64.97 ± 1.80 $\begin{array}{l} {}***\\ \#\#\# \end{array}$	82.65 ± 2.40 $\begin{array}{l} {}***\\ \#\#\# \end{array}$
2.0	47.45 ± 1.87	52.06 ± 1.96	61.78 ± 3.42 ***	78.34 ± 2.74 $\begin{array}{l} {}***\\ \#\#\# \end{array}$	90.76 ± 2.79 $\begin{array}{l} {}***\\ \#\#\# \end{array}$
4.0	61.84 ± 2.01	67.14 ± 3.37	75.69 ± 2.83 ***	90.57 ± 1.28 $\begin{array}{l} {}***\\ \#\#\# \end{array}$	93.96 ± 2.94 $\begin{array}{l} {}***\\ \#\#\# \end{array}$
8.0	74.50 ± 4.17	79.91 ± 2.23	89.21 ± 1.78 ***	95.53 ± 2.06 $\begin{array}{l} {}***\\ \#\#\# \end{array}$	98.10 ± 1.44 $\begin{array}{l} {}***\\ \# \end{array}$

Data are expressed as mean ± standard deviation (mean ± SD, *n* = 4). * Designates significant differences between Pro enantiomers-treated groups and control group. ** *p* < 0.01 and *** *p* < 0.001; # Designates significant differences between *R*-Pro groups and *S*-Pro groups. # *p* < 0.05 and ### *p* < 0.001.

**Table 2:** Effects of Pro enantiomers on MDA, SOD and CAT on TR4.

Item	Unit	Control	Pro Enantiomers (μg/mL)			
			*S*-Pro		*R*-Pro	
			0.4	0.8	0.4	0.8
MDA	nmol/mgprot	29.07 ± 0.61	34.02 ± 0.94 **	59.05 ± 2.06 ***	45.57 ± 0.92 $\begin{array}{l} {}***\\ \#\# \end{array}$	64.24 ± 0.92 ***
SOD	U/mgprot	233.82 ± 13.33	197.71 ± 7.56 *	90.31 ± 2.77 **	129.39 ± 4.66 $\begin{array}{l} {}***\\ \#\#\# \end{array}$	81.69 ± 3.33 $\begin{array}{l} {}***\\ \#\#\# \end{array}$
CAT	U/mgprot	205.80 ± 2.82	196.60 ± 1.69 *	184.18 ± 2.73 ***	180.02 ± 2.92 $\begin{array}{l} {}***\\ \#\# \end{array}$	125.64 ± 2.46 $\begin{array}{l} {}***\\ \#\#\# \end{array}$

Data are expressed as mean ± standard deviation (mean ± SD, *n* = 4). * Designates significant differences between Pro enantiomers-treated groups and control group. * *p* < 0.05; ** *p* < 0.01 and *** *p* < 0.001; # Designates significant differences between *R*-Pro groups and *S*-Pro groups. ## *p* < 0.01 and ### *p* < 0.001.

## Data Availability

Not applicable.

## Supplementary Materials

Supplementary materials can be found at https://www.mdpi.com/article/10.3390/ijms23042352/s1. Reference [54] is cited in the supplementary materials.
