# Supplementary material for: Mechanistic Insights into Stereospecific Antifungal Activity of Chiral Fungicide Prothioconazole against Fusarium oxysporum F. sp. cubense
Source: Int J Mol Sci. 2022 Feb 21;23(4):2352. doi: 10.3390/ijms23042352 (PMC8875126; doi:10.3390/ijms23042352)
Supplement: Supplementary file 1 [file ijms-23-02352-s001.zip › ijms-1580374-supplementary.pdf]

International Journal of *Molecular Sciences*

Supporting Information

Text S1. Fungicidal activity of *rac* prothioconazole (Pro) against TR4

The fungicidal activity of *rac* Pro was determined by calculating the 50% inhibitory dose (EC_{50} value). Petri plates containing PDA medium with different concentrations of triazole fungicides (0.0625, 0.125, 0.250, 0.500, 1.000, 2.000 μg/mL) and control (0.5% DMSO (v/v)) were each inoculated centrally with a mycelial plug (5 mm diameter) taken from the margin of a 7-day-old colony of TR4 grown on PDA medium with 6 replicates. The inoculation was carried out at 28 °C for 7 days in the dark and then the colony diameters were recorded and used to calculate the growth inhibition rates of TR4. As shown in Figure S1, Pro exhibited a significant fungicidal activity against TR4, with half maximal effective concentration (EC_{50}) value of 0.806 μg/mL.

Text S2. Gene expression assay

The RNA content was determined at 260 nm by a spectrophotometer (M2; Molecular Devices, CA, USA), and the purity and quality were evaluated by the 260/280 nm ratios. Synthesis of first-strand cDNA was performed by using a PrimeScript® RT Reagent Kit (TaKaRa, Dalian, China) following the manufacturer's instructions. Primer details are presented in the Table S1 and the melting curve of each primer was analyzed to ensure only one single product was amplified. The housekeeping was verified by using geNorm software, and the results showed that *g6dh* gene did not vary upon Pro enantiomers exposure and therefore was selected as the internal standard.

Text S3. Protein expression and purification

The N1RIS1_FUSC4 gene from *Foc* TR4 was synthesized and its nucleotide sequence was optimized for expression in *E. coli* and engineered to contain 5′ NdeI and 3′ HindIII sites and at the same time a C-terminal hexahistidine tag in order to facilitate the subsequent purification process (Sangon Biotech, Shanghai, China). N1RIS1_FUSC4 protein was produced in BL21 (DE3) cells (Transgene, Peking, China) at 20 °C by using the pET-28a(+). Isopropyl-D-thiogalactoside (IPTG) was used to induce protein expression when OD600 reached to 0.6. Cells were harvested by centrifugation (5000 rpm, 5 min), resuspended with wash buffer (20 mM Na_3PO_4, 500mM NaCl, pH 7.4) and subjected to ultrasonic treatment. The clear lysate was collected by centrifugation (15000 rpm, 30min). Commercially available Ni^+ columns (HisGraviTrap™ HP column (GE Healthcare)) was used to purify the expressed histidine-tagged recombinant protein according to the manufacturer's instructions. The protein elution was collected under native conditions in an elution buffer (20 mM Na_3PO_4, 500 mM NaCl with increasing imidazole concentration). The eluates were then analyzed by 12% SDS-PAGE followed by Coomassie Brilliant Blue staining and western blot analysis, and the protein concentration was determined using the BCA protein assay kit.

Text S4. Binding affinity assay

The expressed recombinant N1RIS1_FUSC4 protein was immobilized on the CM5 sensor chip surface using the standard amine-coupling procedure. In brief, with N-ethyl-N0-(dimethylaminopropyl) carbodiimide/N-hydroxysuccinimide (EDC/NHS) using 10 mM NaAc (pH 5.0) as a coupling buffer and HBS-EP (0.01 M HEPES, 0.15 M NaCl, 0.003 M EDTA, 0.05% v/v surfactant P20, pH 7.4) as a running buffer. N1RIS1_FUSC4 protein was amine coupled in the second flow channel and the first flow channel was left unmodified. The assay was performed by priming the system 3 times with running buffer and by 5 start-up cycles with injection of running buffer. Assay solutions of Pro enantiomers were prepared by being dissolved in HBS-EP containing 5% DMSO from 3.125 μM to 50.00 μM (3.125 6.250, 12.50, 25.00, 50.00 μM) respectively. The assay solutions were injected at

a flow rate of 30 μL/min under the conditions of HBS-N containing 5% DMSO and 0.05% surfactant P20 as a running buffer. For each test chemical, the association phase of the SPR measurement was 120 s and the buffer flow continued to allow a dissociation time of 600 s followed by an extra wash of the flow system of 50% DMSO. DMSO calibration plot was constructed to correct bulk refractive index shifts [54] by injecting buffer samples containing 4.5-5.8% DMSO. The kinetic constants were obtained by fitting the experimental data to a reversible 1:1 bimolecular interaction model included in the Biacore T200 evaluation software 3.0 from the instrument manufacturer.

Table S1. Primer sequence used in the present study.

Gene ID & Description	Forward Primer Sequence (5'-3')	Reverse Prime Sequence (5'-3')
g6dh	ATATTCCCCGAAACGAGCTT	ATGCTGAGACCAGGCAACTT
cyp51	CCCCACTGGAAACGACTTCA	AGCATGTTTGCTACGCAAGG

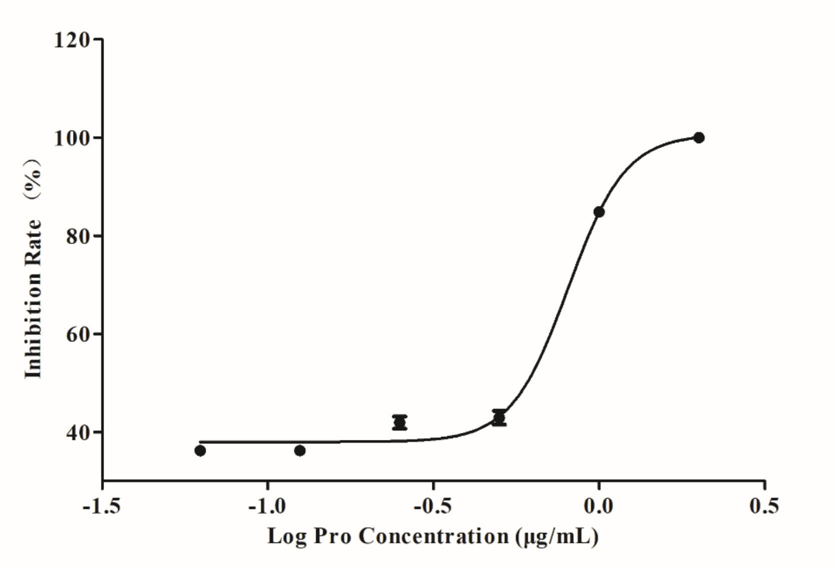

Figure S1. The inhibition rate (%) of *Foc* TR4 after 7 days growth on agar plates, supplemented with increasing amounts of *rac* Pro. The concentration-response curve exhibited sigmoid characteristics with a unitary slope. The EC_{50} concentration for *rac* Pro was equal to 0.806 μg/mL.

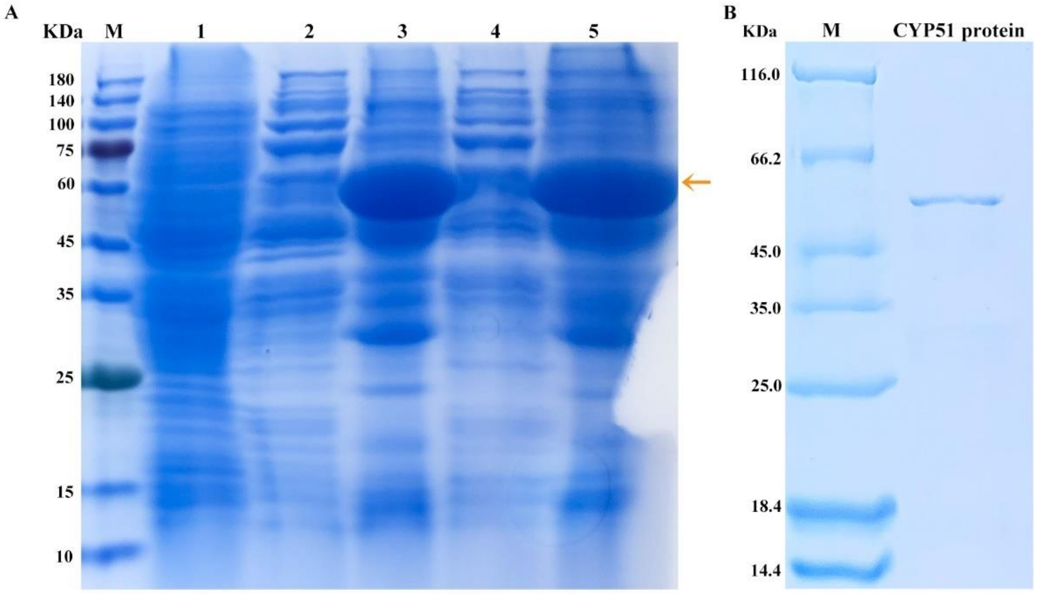

Figure S2. Expression and purification of the N1RIS1_FUSC4 protein from TR4. (**A**) SDS-PAGE analysis of IPTG-induced expression of recombinant N1RIS1_FUSC4 in *E. coli*. M: marker processed; 1. Non-IPTG-induced; 2. Supernatant of cell debris with IPTG induction at 20 °C; 3. Precipitates of cell debris with IPTG induction at 20 °C; 4. Supernatant of cell debris with IPTG induction at 37 °C; 5. Precipitates of cell debris with IPTG induction at 37 °C; Yellow arrow indicates N1RIS1_FUSC4 protein. (**B**) SDS-PAGE for purified N1RIS1_FUSC4 with Ni^{+} columns. M: marker processed; CYP51 protein. purified expression product N1RIS1_FUSC4.

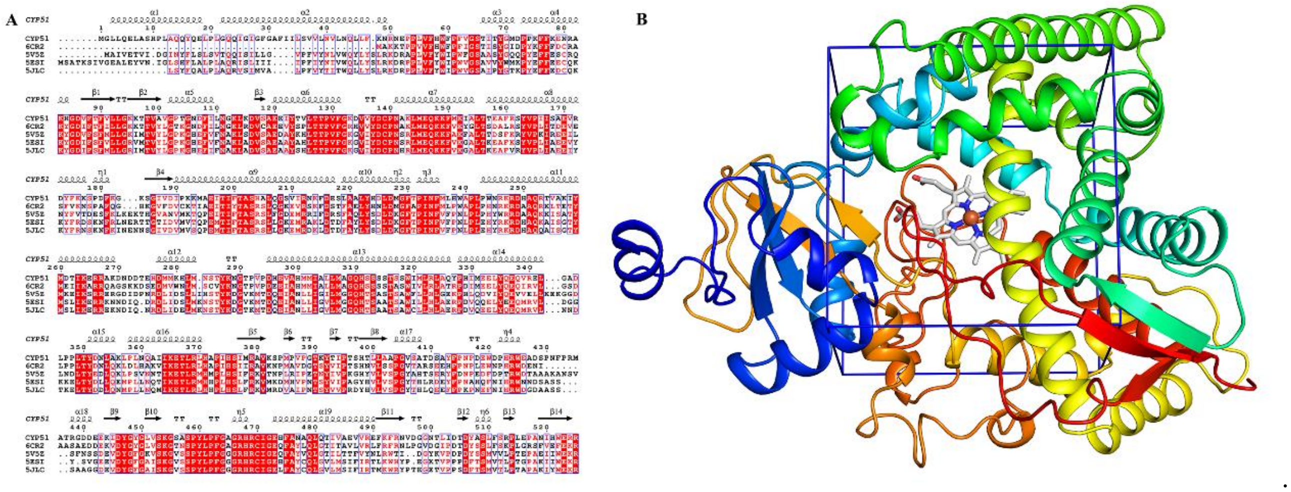

Figure S3. Homology modeling of CYP51 from TR4. (**A**) Sequence alignment of CYP51 proteins from TR4, 5V5Z, 5JLC and 5ESI through multi-template modeling. (**B**) Model of the CYP51 protein.
